# Viral Families With Pandemic Potential

**DOI:** 10.1093/ofid/ofaf306

**Published:** 2025-05-26

**Authors:** Amesh Adalja, Thomas Inglesby

**Affiliations:** Johns Hopkins Center for Health Security, Johns Hopkins Bloomberg School of Public Health, Baltimore, Maryland, USA; Johns Hopkins Center for Health Security, Johns Hopkins Bloomberg School of Public Health, Baltimore, Maryland, USA

**Keywords:** Pandemic Preparedness, Respiratory Viruses

A major challenge of pandemic preparedness is how to anticipate and prepare for future pandemic threats among the wide range of viral threats that can infect humans. Fortunately, only a subset of the 25 viral families that can infect humans have both the capability of widespread respiratory transmission in humans or animals, a prerequisite for pandemic-causing capability, as well a lack of medical countermeasures (MCMs) to prevent and treat the key viral species within them. Orthopoxviruses, for example, clearly have historically shown the capacity to cause terrible pandemics in humans, but vaccines and antivirals have been developed and stockpiled for this viral family that are cross-protective against multiple family members (unlike the case for the pandemic-causing orthomyxoviruses and coronaviruses). Therefore, for the purposes of identifying novel rapid-onset novel pandemics, they are not listed here as a viral family of pandemic potential with unmet research and development (R&D) requirements, although there is clear need for improved MCMs to both of these viral families.

Prior to coronavirus disease 2019 (COVID-19), most pandemic preparedness efforts, when they did occur, focused almost exclusively on influenza viruses. Pandemic preparedness was considered to be nearly synonymous with influenza preparedness. This has basis in experience as the only occurring pandemics for almost a century (spanning from at least 1918 to 2009) were all caused by influenza A viruses. However, a number of events of the past 20 years have shown the capacity of Coronaviridae to cause major epidemics and pandemics: severe acute respiratory syndrome coronavirus (SARS-CoV) 1 in 2003, Middle East respiratory syndrome coronavirus in 2012, and, most recently and obviously, SARS-CoV-2 in 2019–2020. It is critical now to plan for other viral threats that can cause huge epidemics or pandemics.

We were among the first to publish on the characteristics of pandemic pathogens, and in that effort, we emphasized conceptualizing the problem by putting specific focus on viral families of greatest pandemic potential, particularly families for which no MCMs were yet developed [1, 2]. There have been subsequent projects to categorize viral families with greatest pandemic potential; those that have been published have cast a wider net and have so far used inclusion criteria that are significantly broader than what we have used for the categorization in this work [3–5]. The benefit of the other organizations’ broader inclusion criteria is that they have encompassed substantially more viral families; the downside of such broader inclusion criteria is that it can include viral families with little evidence of their prospect of generating a new pandemic. This essay proposes a more tailored, focused approach to preparedness based on both pathogen pandemic potential and R&D.

Drawing on that prior work, here we describe with more detail the viral families that should be prioritized as those with greatest pandemic potential given their capacity for widespread respiratory transmission and the absence of MCMs: Orthomyxoviridae, Coronaviridae, Paramyxoviridae, Picornavirdae, Pneumoviridae, and Adenoviridae. These viral families all include viral species that have the capacity for efficient human-to-human spread via the respiratory route, seasonal endemic members, and zoonotic analogues. As we have argued in prior work [1], in the modern era, the respiratory mode of transmission is the distinguishing characteristic between epidemic and worldwide acutely disruptive pandemic viruses. The human immunodeficiency virus, by contrast, given its mode of transmission, did not have the same propensity for explosive growth. The special status of respiratory infectious diseases derives chiefly from the difficulty of public health interventions to arrest their spread and the potential for asymptomatic or minimally symptomatic contagiousness. Given that the great majority of new infectious disease threats are spillovers from animal viruses, it is likely that pandemic threats will spill over from animal viruses as well. SARS-CoV-2 is a case in point. However, the spillovers most likely to cause pandemic threats will emerge from a zoonotic virus that is a member of the respiratory viral families whose other members have shown the capacity to cause extensive respiratory infection in humans.

There are biological and medical characteristics for each of the 6 viral families that make them the most likely families to generate future pandemic viruses. Chiefly, they each have viral species within them that spread efficiently between humans. Within these viral families there is also a lack of MCMs for many of the serious potential viral threats. No other viral families so starkly possess these combined characteristics [1]. The lens we apply is in distinction to some other groups that use more inclusive criteria for pandemic capacity.

While the potential pandemic properties of Orthomyxoviridae and Coronaviridae are widely appreciated, it is valuable to highlight some of the characteristics of the other 4 less appreciated viral families with greatest pandemic potential [6] (see Table 1).

**Table 1.** Characteristics of 4 of Viral Families With the Greatest Pandemic Potential

Family	Characteristics
Picornaviridae [7]	Nonenveloped viruses (and thus efficiently transmitted via fomites) that efficiently spread via respiratory, fecal-oral, and fomite routes; members include those that are the most common cause of community-acquired pneumonia (rhinovirus), causes of paralytic illness (EV-D68), and encephalitis (EV-A71); zoonotic members pose spillover risks; viruses were not suppressed like other viruses during the COVID-19 social distancing era, attesting to the resiliency of this family [8]; there are no antivirals or vaccines (except polio and hepatitis A vaccines, which have little bearing on other picornavirus members; EV-A71 vaccines are available in non-US countries
Paramyxoviridae [9]	Major cause of upper and lower respiratory tract infections (eg, PIV), as well as those that cause more severe manifestations, such as encephalitis; multiple zoonotic counterparts (eg, Nipah) that infect humans and are endemic in other species (eg, zoonotic morbilliviruses); members with extremely high transmissibility (ie, measles); no antivirals or vaccines (except for measles and mumps)
Adenoviridae [10]	Efficiently transmitting viruses that can cause explosive outbreaks with severity (eg, Ad14 and Ad7); multiple zoonotic counterparts; military-only vaccine for 2 serotypes; no antivirals
Pneumoviridae [11, 12]	Efficiently transmitting viruses that can cause considerable disease and death; member metapneumovirus is a zoonotic virus that has become endemic in humans and is a major cause of respiratory illness, and other zoonotic metapneumoviruses exist; preventive MCMs only for RSV (vaccines, monoclonal antibodies); limited-use toxic antiviral (ribavirin); no MCMs for metapneumovirus

Abbreviations: COVID-19, coronavirus disease 2019; MCMs, medical countermeasures; PIV, Parainfluenza virus; RSV, respiratory syncytial virus.

In conclusion, prioritizing the viral families with greatest pandemic preparedness should be a strong driver for focusing the research, development, and manufacturing agenda for preparedness in the US and globally. This preparedness work should include the following:

1. Developing prototype vaccine and monoclonal antibody candidates to at least phase 2A or phase 2B for the viral families with pandemic potential as well as vaccines for endemic viral species of these viral families (which is not only valuable in treating endemic disease but also provides critical immunological data for treatment and product development for pandemic members of the family). Demonstration of the capacity to manufacture these biological products at scale should also be a key part of this work.
2. Developing antiviral candidates to phase 2A or B for the viral families with pandemic potential or through approval if there is a current clinical need for an existing disease in that viral family. In addition, the broad-spectrum antiviral ribavirin should be taken through formal approval processes for its oral and intravenous formulation for respiratory syncytial virus and other viruses for which it is effective to facilitate greater ease of use in the event of a pandemic in which it may be useful.
3. Promoting platforms that are used to develop the successful, specific prototype vaccine, monoclonals, and antivirals, as an important part of the preparedness portfolio.
4. Enhancing diagnostics, including home and point-of-care devices for pandemic viral families beyond the versions that exist for influenza, SARS-CoV-2, and respiratory syncytial virus.

Of course, other viral families have the potential to cause serious illness, endemic disease, and high-consequence local or larger epidemics, and they merit scientific attention and MCM development commensurate with their substantial public health impact. However, R&D and manufacturing being pursued with the goal of preparing the country and the world for the next pandemic should first prioritize viral families with greatest pandemic potential. We are encouraged by the UK government's priority pathogen tool, which includes paramyxovirus and picornaviruses [13]. There are clear and tractable MCM development goals that should be pursued for these viral families. Accomplishing this work would substantially strengthen our collective capacity to diminish the suffering, illness, and deaths associated with future pandemics.

## Note

***Potential conflicts of interest.*** All authors: No reported conflicts.
